# *Larsenianthus*, a new Asian genus of Gingers (Zingiberaceae) with four species

**DOI:** 10.3897/phytokeys.1.658

**Published:** 2010-11-01

**Authors:** W. John Kress, John D. Mood, Mamiyil Sabu, Linda M. Prince, Santanu Dey, E. Sanoj

**Affiliations:** 1 Department of Botany, MRC-166, National Museum of Natural History, Smithsonian Institution, P.O. Box 37012, Washington, DC 20013-7012 U.S.A.; 2 Lyon Arboretum, University of Hawaii, 3860 Manoa Road, Honolulu, HI 96822 U.S.A.; 3 Department of Botany, University of Calicut, 673635 Kerala, India; 4 Rancho Santa Ana Botanic Garden and Claremont Graduate University, 1500 North College Avenue, Claremont, CA 91711-315 U.S.A.; 5 Aaranyak, Samanwoy Path, #50, Beltola, Guwahati-28, Assam, India; 6 Department of Botany, University of Calicut, 673635 Kerala, India

**Keywords:** Bangladesh, conservation status, DNA barcodes, *Hitchenia*, India, Myanmar, phylogeny, taxonomy

## Abstract

Larsenianthus W. J. Kress & Mood, **gen. nov.** is described with one new combination and three new species. Larsenianthus careyanus (Benth.) W. J. Kress & Mood, **comb. nov.**, is widespread in India and present-day Bangladesh; Larsenianthus wardianus W. J. Kress, Thet Htun & Bordelon, **sp. nov.**, is from upper Myanmar in Kachin State; Larsenianthus assamensis S. Dey, Mood, & S. Choudhury, **sp. nov.**, is restricted to Assam, India; and Larsenianthus arunachalensis M. Sabu, Sanoj & T.Rajesh Kumar, **sp. nov.**, has only been found in Arunachal Pradesh, India. A phylogenetic analysis using the plastid *trnK* intron and nuclear ITS DNA sequence data indicates that the four species of Larsenianthus form a monophyletic lineage that is sister to Hedychium, a geographically widespread genus of about 50 species in tribe Zingibereae of subfamily Zingiberoideae. A dichotomous key and three-locus DNA barcodes are provided as aids for the identification of the four species of Larsenianthus.

## Introduction

The classification of the family Zingiberaceae continues to be refined (Kress et al. 2002, 2007, Harris et al. 2006) and new taxa are still being discovered and described (e.g., Lý et al. 2010). Recent field work in South Asia has not only uncovered taxa new to science, but also provided new insights into our understanding of generic boundaries and species definitions (Sabu 2006). Here we clarify the generic placement of a species first recognized over 125 years ago and describe a new genus of gingers with three additional new species.

In 1835 Nathaniel Wallich applied the name Hitchenia glauca to a ginger that he had listed earlier in his catalogue (Wallich 1832) as Curcuma glaucophylla Wall. (no. 6594). Another species listed in his catalogue as Curcuma careyana Wall. (no. 6595) was later moved to the genus Hitchenia by Bentham, who neither described its morphology nor provided any reason for the new generic placement (Bentham and Hooker 1883). A third species, which had previously been listed as Curcuma caulina J. Graham (Graham 1839), was added to the genus Hitchenia as Hitchenia caulina (J.Graham) Baker (Baker 1892). In that publication Baker circumscribed the genus Hitchenia and provided more detailed descriptions of the other two species, Hitchenia glauca and Hitchenia careyana. However, he realized the taxonomic problems that existed and stated that the latter species “..resembles Hitchenia glauca in habit, but differs so much in structure that probably it should form a different genus…” (Baker 1892).

In Wallich’s 1835 original application of the generic name, the genus honored Mr. Thomas Hitchin of Norwich, England, who was a gardener and distributor of rare plants in the 1810–1830s (Noltie 2005). Unfortunately Wallich misspelled Thomas Hitchin’s name when he published the new genus as Hitchenia. Horaninow (1862) changed the name back to Hitchinia to correct Wallich’s mistake, but this nomenclatural revision was not recognized by later botanists. In fact Bentham and Hooker (1883) mentioned Horaninow’s corrected generic spelling as an error and preferred to use Hitchenia.

**Plate 1.**
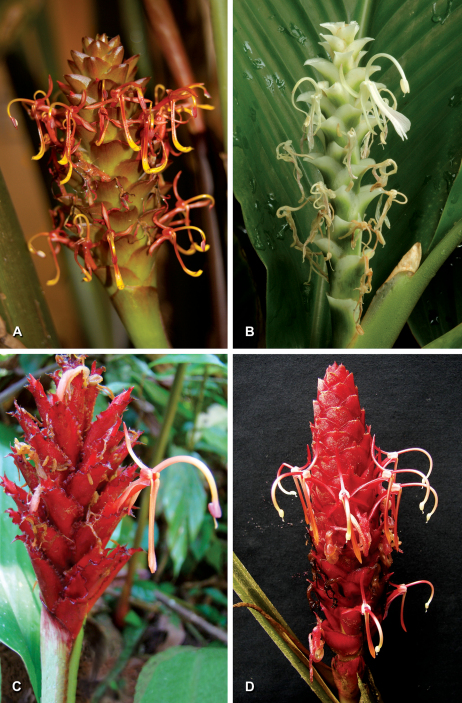
Inflorescences with flowers of **A** Larsenianthus wardianus W. J. Kress, Thet Htun & Bordelon **B** Larsenianthus careyanus (Benth.) W. J. Kress & Mood **C** Larsenianthus assamensis S. Dey, Mood, & S. Choudhury **D** Larsenianthus arunachalensis M. Sabu, Sanoj & T.Rajesh Kumar. All photos by the authors.

Since the time of Baker, little progress has been made in understanding the taxonomic status of Hitchenia until the publication of a new classification of the Zingiberaceae by Kress et al. (2002). Hitchenia glauca, which had been once more discovered in Myanmar after many years of obscurity, was included in the molecular phylogenetic study of the family and shown to be closely related to several species of Curcuma It was tentatively placed in Curcuma Group I (Kress et al. 2002). At about the same time as the publication of the new classification of the family, living specimens of Hitchenia careyana (Plate 1B) were collected near the type locality of Sylhet (formerly Silhet) in Bangladesh (M. Collins, pers. com.). From a comparison by the authors of these living collections to material of Hitchenia glauca, it became clear that these two taxa were only superficially similar and probably not at all closely related to each other. For this reason it appeared that a new generic name was needed for Hitchenia caryeana.

In 2002, as part of a survey of the gingers of Myanmar, one of us (WJK) collected specimens of an unknown ginger near Myitkyina, in Kachin State, which closely resembled Hitchenia careyana, but were distinctive in the inflorescence color and orientation (Plate 1A). In 2008 another of us (SD) discovered an unusual ginger during field work in southern Assam. Surprisingly, the flowers closely resembled those of both the former Hitchenia caryeana and the newly collected material from Myanmar (Plate 1A-C). Finally, only a few weeks later, a fourth species of ginger with the same distinctive inflorescence and floral morphology was recognized by the third author (MS) of this paper whose botanical team had just returned from a survey of plants in northeast India (Plate 1D).

After studying both living specimens and preserved collections of all four taxa, the accumulated research data were sufficient to warrant the circumscription of a new genus. The long exserted and arched style (with included filament) of the flower resembles a similar floral structure found in genera such as Hedychium, Globba, and Pommereschea. In order to obtain evidence of the origin of this floral characteristic in the new genus, to provide an independent test of the evolutionary relatedness of these four species, and to determine their position in the evolution of the family Zingiberaceae a molecular phylogenetic investigation was undertaken (see below). All data suggested that these four species should be placed in a new genus, which we have named Larsenianthus, allied to Hedychium.

With regards to the remaining species in the genus Hitchenia, Leong-Škorničková et al. (2007) in a study of chromosome number and genome size variation in the gingers demonstrated that one species, Hitchenia caulina, would be better placed in the genus Curcuma as initially proposed by Graham (1839). Eventually some taxonomists may prefer to subsume both species of Hitchenia into Curcuma.

Below we provide descriptions of the new genus and the four included species. Plant measurements were for the most part recorded from living material. Methods and results of the molecular phylogenetic analysis, GenBank accession numbers for a three-locus DNA barcode for each species, and assessments of the conservation status of all species of Larsenianthus are also provided.

## Phylogenetic evidence and DNA barcode markers

### Materials and methods

#### DNA extraction, amplification, and sequencing.

Total genomic DNAs were extracted for a representative sample of each species of Larsenianthus described herein (Table 1) using a modified CTAB protocol. Nucleic acid fragments for *trnK* and ITS as phylogenetic markers were amplified using custom primers and Promega Go Taq Flexi under standard cycling conditions, and cleaned using an abbreviated PEG/NaCl procedure as described in Kress et al. (2002). Amplification of *rbcL* and the *trnH-psbA* spacer region as DNA barcoding loci used published primers under standard conditions (Kress and Erickson 2007). The *matK* DNA barcode locus was isolated from the *trnK* intron sequences that were generated for the phylogenetic analyses. Fragments were fluorescently labeled using Applied Biosystems (Foster City, California, USA) Big-Dye v3.1 (1/8 concentration) chemistry Terminator Cycle Sequencing Ready Reaction Kit following AB protocol for a 3130xl Automated DNA Sequencer. DNA fragments were compiled and edited in Sequencher 4.9 (Gene Codes Corp., Ann Arbor, Michigan, USA).

#### Phylogenetic analyses.

Newly generated sequences of the *trnK* intron and ITS were added to a reduced taxon version of the Kress et al. (2002) data matrix, which included representatives of all subfamilies of the Zingiberaceae. Siphonochilus was designated as the outgroup based on results of the prior study (Kress et al. 2002). The data matrix was realigned using MUSCLE (Edgar 2004) as implemented in Geneious Pro v4.8.5 (Biomatters Ltd., Auckland, New Zealand; www.geneious.com) followed by minor manual adjustment with indels treated as missing data.

*Maximum Parsimony*. Separate and combined Fitch parsimony analyses of one thousand random sequence addition replicates with tree bisection and reconstruction (TBR) branch swapping, holding four trees, saving all shortest trees were conducted in PAUP *4.0b10 (Swofford 2002). Branch support was calculated based on 1,000 bootstrap pseudoreplicates (BS) of 100 random addition replicates (holding four trees, TBR branch swapping, saving 10 trees per replicate) to maximize the accuracy of the estimation while minimizing analysis time.

*Likelihood*. Bayesian analyses were conducted in MrBayes (Huelsenbeck and Ronquist 2001, Ronquist and Huelsenbeck 2003) to estimate branch support using three replicates of five million generations (sampling every 100 generations), running four chains. The *trnK* dataset was partitioned into three regions (*trnK*5’ IGS, *matK*, and *trnK*3’IGS) and the combined dataset was partitioned into four regions (*trnK* as described above, plus ITS). Appropriate burn-in (number of generations discarded prior to calculation of posterior probability [PP]) for each analysis was determined based on a standard deviation of split frequencies (discarding all trees prior to stabilization below a standard deviation >0.01). Burn-in times for each data matrix are available from the senior author.

### Results

#### ITS.

Analyses of the ITS data matrix produced 23 shortest trees of 1,011 steps based on 268 parsimony informative characters (results not shown). Trees fell into three distinct tree islands, one of 21 trees, and two of one tree only. Differences between these three tree islands were minor. In all shortest trees, the four species of Larsenianthus form a monophyletic clade with moderate to strong support (BS=89%; PP=1.00), and are sister to a monophyletic clade of Hedychium (BS=100%; PP=1.00). The sister relationship is also supported although less strongly than the reciprocal monophyly of the genera if only parsimony is considered (BS=68%; PP=1.00).

#### trnK intron.

*trnK* intron analyses produced over 100,000 shortest trees of 552 steps based on 328 parsimony informative characters (results not shown). Relationships were generally less well resolved and less strongly supported than with the ITS data, however there were missing data for part of the 5’ *trnK* sequences for two of the Larsenianthus samples. Monophyly of Hedychium was moderately supported by parsimony (BS=84%) and strongly supported by Bayesian methods (PP=1.00). The majority rule consensus tree recovered a monophyletic Larsenianthus, but not the strict consensus tree. Three of the four species of Larsenianthus formed a monophyletic group with low to moderate support (BS=65%; PP=0.96). Similarly, a sister relationship between the two genera was not recovered.

#### Combined ITS & trnK.

**Figure 1.**
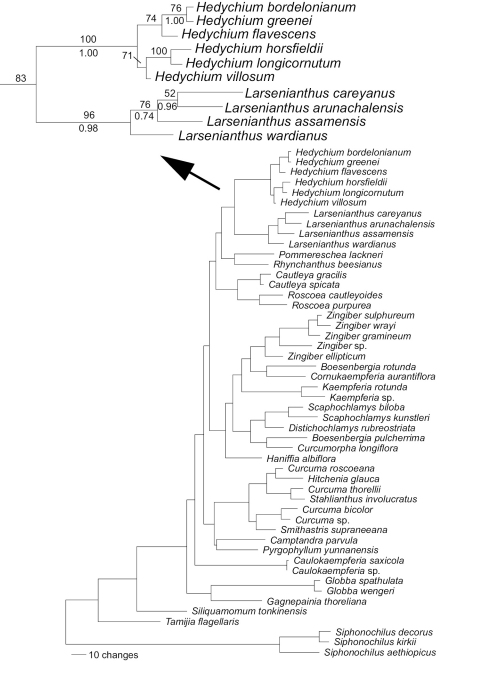
Molecular phylogeny of the Zingiberaceae indicating the placement of Larsenianthus within the Zingiberoideae in the combined ITS/*trnK* analysis. Tree length (excluding uninformative characters) = 1,590 steps, Consistency Index = 0.5314, Retention Index = 0.6809, Rescaled Consistency Index = 0.3619. Bootstrap numbers are provided above the branch, Posterior Probabilities below.

The combined data analyses produced 18 shortest trees of length 1,590 steps (Fig. 1). Analyses utilized 585 parsimony informative characters. Larsenianthus and Hedychium were both monophyletic with strong branch support (Hedychium: BS=100%; PP=1.00; Larsenianthus: BS=96%; PP=0.98); the sister relationship between the two genera was confirmed with moderate support (BS=83%; PP=0.92; Fig. 1).

#### DNA barcodes.

The DNA barcodes that were generated for the three barcode loci, *rbcLa*, *matK* and *trnH-psbA*, (Kress and Erickson 2007, CBOL Plant Working Group 2009) provide unique genetic identifiers for each of the four species of Larsenianthus. GenBank accession numbers for sequences of each DNA barcode locus for each species are provided (Table 1).

**Table:** **Table 1.** List of species of Larsenianthus with vouchers (herbarium location) and GenBank accession numbers for DNA barcodes and gene sequences used in the phylogenetic analyses.

*Taxon*	*Voucher*	*ITS*	trnK (including matK)	rbcLa	trnH-psbA
Larsenianthus wardianus	W. J. Kress 10-8750 (US)	HM771392	HM771404	HM771400	HM771396
Larsenianthus careyanus	W. J. Kress 03-7403 (US)	HM771393	HM771405	HM771401	HM771397
Larsenianthus assamensis	S. Dey 1012 (CAL)	HM771395	HM771407	HM771403	HM771399
Larsenianthus arunachalensis	Sanoj & Rajesh Kumar 105640 (CAL)	HM771394	HM771406	HM771402	HM771398

### Conservation status

The overall distribution of the genus covers a large area of sub-Himalayan forests in northeastern Bangladesh, the Indian states of Meghalaya and Assam, the northern border of Arunachal Pradesh, and into upper Myanmar. Larsenianthus careyanus is the most widespread of the species and by IUCN guidelines (IUCN Standards and Petitions Subcommittee 2010) we categorize it as *Near Threatened* (NT) because of the gradual decline of the habitats it occupies and the small number of recent collections. The other three new species are only known from their type localities. Until more collections are made of these three species, we estimate a provisional conservation status of *Critically Endangered* (CR: B1ab(iii) + B2ab(iii)).

### Taxonomy

#### Larsenianthus

W. J. Kress & Mood gen. nov.

urn:lsid:ipni.org:names:77107682-1

**Figure 2. F2:**
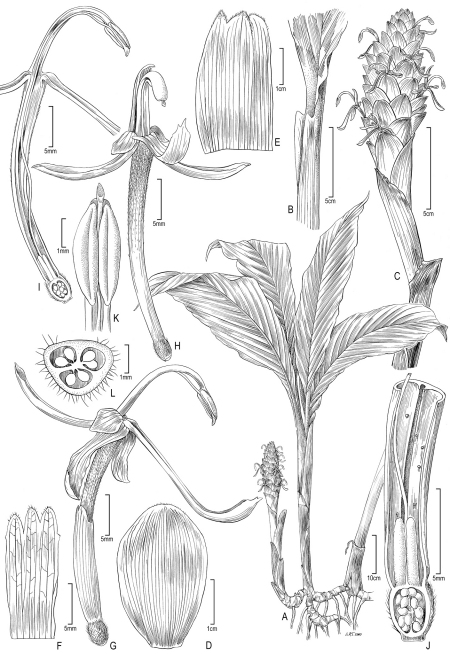
Larsenianthus wardianus W. J. Kress, Thet Htun & Bordelon. **A** overall habit **B** leaf base with petiole and ligule **C** inflorescence **D** inflorescence bract **E** bracteole **F** calyx, spread open **G** flower, lateral view **H** flower, front view **I** flower, semi-lateral view with corolla tube cut away to show epigynous nectaries and style **J** base of flower, cut-way view to show style, and ovary **K** anther with slightly protruding stigma **L** ovary, transverse section. Line drawing by Alice Tangerini from plants in cultivation; Botany Research Greenhouse Accession #02-7054and US National Herbarium voucher W. J. Kress 10-8750.

**Figure 3. F3:**
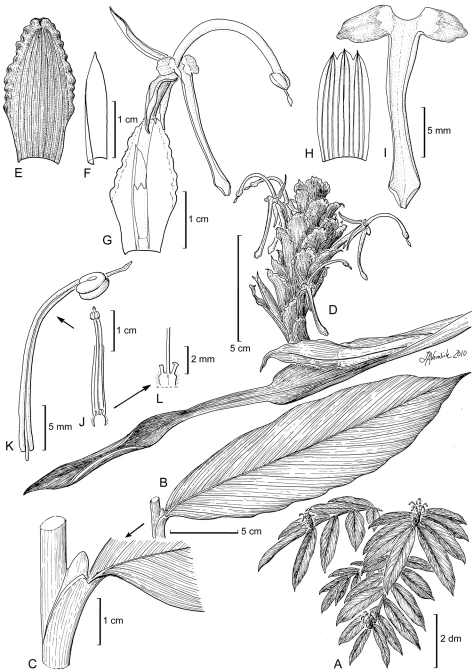
Larsenianthus assamensis S. Dey, Mood, & S. Choudhury. **A** overall habit **B** leaf, entire **C** leaf base with petiole and ligule **D** inflorescence **E** inflorescence bract **F** bracteole **G** bract and flower, semi-lateral view **H** calyx, spread open **I** labellum with attached lateral staminodes **J** stamen showing base of floral tube and the epigynous nectaries **K** filament and anther with protruding stigma **L** epigynous nectaries and style. Line drawing by Linda Van Vorobik from plants in cultivation; voucher S. Dey 1012.

**Figure 4.**
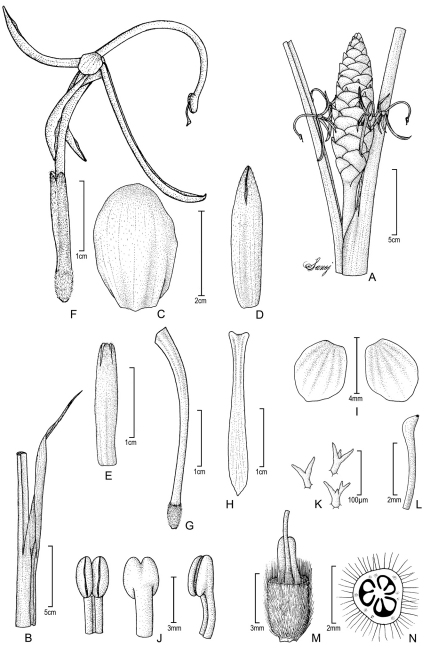
Larsenianthus arunachalensis M. Sabu, Sanoj & T.Rajesh Kumar. **A** inflorescence **B** apical part of leaf sheath with ligule and petiole **C** inflorescence bract **D** bracteole **E** calyx **F** flower, lateral view **G** floral tube **H** labellum **I** lateral staminodes **J** anther, front, back and lateral views **K** unicellular branched hairs inside corolla tube **L** stigma and upper portion of style **M** base of flower, cut-way view to show style and epigynous nectaries **N** ovary, transverse section. Line drawing by E. Sanoj from plants in cultivation; voucher Sanoj & Rajesh Kumar 105640.

[Fig F2] [Fig F3] Figs. 2–4 Plate 1A-D 

##### Latin

*Zingiberacearum tribus Zingiberearum genus novum* Hedychio *J. Koenig affine, a quo staminodiis lateralibus parvis auriculatis, labello angusto elongato marginibus incrassatis carinaque centrali et filamento valde arcuato differt*.

##### Type.

Larsenianthus careyanus (Benth.) W. J. Kress & Mood, comb. nov., Hitchenia careyana Benth., Gen. Pl. 3: 643. 1883.

##### Description.

Evergreen, rhizomatous, terrestrial herbs, clumping with 10–20 shoots per plant, 1–2.5 m tall, plane of distichy of leaves parallel to rhizome, 2–12 leaves per shoot, alternate, sessile to petiolate. Inflorescence terminal on leafy shoot or basal on leafless shoot, pedunculate; bracts basally attached, reflexed or adpressed, spirally arranged and imbricate, 35–80 per inflorescence, not pouched, adventitious plantlets sometimes produced in sterile bracts at base of inflorescence; flowers mature from base to apex of inflorescence. Bracteoles variable in size, the inner one largest, not tubular. Flowers conspicuous, in cincinni of 2–6 flowers or rarely reduced to one flower; calyx tubular, tri-dentate, shorter than the corolla; floral tube long and curved, exserted well beyond the bract, corolla lobes subequal with dorsal lobe slightly larger than laterals, apex opening oblong, bordered on the two lateral sides with a thickened and rounded margin formed by the base of the lateral staminodes and the labellum; lateral staminodes small, bowl-shaped, reflexed; labellum narrow at the base, widening towards the apex, elongate, oblanceolate or spatulate, basal margins thickened, raised with center channeled or ‘v’ in cross-section, apex dentate or entire; fertile stamen long and arched over the labellum, anther oblong, without a crest, thecae dehiscent for full length; epigynous glands linear; style with stigma protruding beyond anther thecae; ovary trilocular, fruit an oblong capsule, two-layered with the outer splitting into three coiled sections, the inner forming an arillate membrane covering the 1–10 seeds.

##### Discussion.

Larsenianthus is a distinctive genus in the tribe Zingibereae. Some similarities exist with the genera Hedychium, Globba and Pommereschea in the vegetative parts, the bract orientation, and the long filament. The uniqueness of the genus and its close relationship to Hedychium is confirmed by phylogenetic analyses of DNA sequence data (Fig. 1). However, the combination of unique features including the narrow, elongate labellum with slightly thicken edges and a deeply channeled center combined with the small, cup-shaped lateral staminodes and strongly arched filament clearly distinguish this genus from others in the tribe. Two additional traits that characterize at least two of the four species in the genus (Larsenianthus assamensis and Larsenianthus careyanus) are the unusual white capsular fruit with a gelatinous fused arillate structure that encases the seeds and the multiple adventitious plantlets that form in the axils of the sterile bracts at the base of the inflorescence.

##### Etymology.

This new genus honors Dr. Kai Larsen, Professor of Botany Emeritus, Aarhus University, Denmark, for his many years of dedicated efforts in the education of botanists worldwide and for his profound achievements in the taxonomy of the family Zingiberaceae, especially in the flora of Thailand.

##### Key to species of Larsenianthus

.

**Table:** 

1b.	Inflorescence terminal on a leafy shoot	2
2a.	Inflorescence bracts green and white, 4–6 flowers per bract	Larsenianthus careyanus
2b.	Inflorescence bracts red, 1–3 flowers per bract	3
3a.	Margins of inflorescence bracts corrugate and denticulate, 1 flower per bract	Larsenianthus assamensis
3b.	Margins of inflorescence bracts entire and smooth, 2–3 flowers per bract	Larsenianthus arunachalensis

#### Larsenianthus wardianus

W. J. Kress, Thet Htun & Bordelon sp. nov.

urn:lsid:ipni.org:names:77107683-1

Fig. 2 Plate 1A 

##### Latin

*Nova species* L. careyano *affinis, a quo minore statura, congestis foliis, inflorescentia radicali in bractea sine foliis, cum paucis floribus in unaquisque bractea differt*.

##### Type.

**Myanmar:** Kachin State: Myitkyina Township, NW section of Pidaung Wildlife Sanctuary, approx. 15 km. from Myitkyina, 25°34'52" N, 97°14'56" E, understory of evergreen forest. 22 February 2002. W. J. Kress, Thet Htun, M. Bordelon, and Khin Maung Ha 02-7054 (living plant only for cultivation). Plants of #02-7054 cultivated at the Smithsonian Botany Research Greenhouses. 14 April 2010. W. J. Kress 10-8750 (holotype: US!; isotypes RAF!, E!).

##### Description.

*Evergreen herb*, medium-size to 125 cm tall; rhizome to 2.0 cm in diameter, fibrous, aromatic, inner color white; tubers present. *Leafy shoots* 10–20 per plant, erect, densely clumped. *Leaves* 4–5 per shoot, to 118 cm in total length; basal leaf sheaths reddish and glabrous, ca. 30 cm in length × 5 cm in width; sheathing petioles to 52 cm in length × 1.5 cm in diameter, glabrous, green and clasping stem, margin slightly revolute; ligule small, 10 mm in length × 1 mm in width, not lobed, truncate on petiole, papery and ephemeral disappearing in mature leaves; lamina 67 cm in length × 14 cm in width, ovate, green and glabrous above, lower midrib green and glabrous, base long attenuate onto petiole, slightly subequal, apex acuminate, adaxial surface dark green. *Inflorescence* basal on leafless shoot, erect to 33 cm in height; peduncle 20 cm in length × 1.0–1.5 cm in diameter, glabrous, lower sheaths red, upper sheaths green; spike ovoid, 6–10 cm in length × 4–5 cm in diameter; rachis straight; inflorescence bracts about 40 per inflorescence, 1–2 lower bracts sterile, bracts 3.7 cm in length × 2.4 cm in width at base of inflorescence, 2.0 cm in length × 1.8 cm in width distally, spirally arranged and imbricate, not pouched, held at 40° from vertical axis, glabrous, bright green with reddish apex, margins smooth; bracteoles not tubular, 2.1 cm in length × 1.6 cm in width, glabrous, pale white with reddish apex. *Flowers* conspicuous, 3–4 per bract; calyx tubular, 1.5 cm long, tri-lobed with central lobe shortest, pale yellow to pink; floral tube 3.2 cm in length × 2.0 mm in diameter, reddish pink, externally glabrous with scattered unicellular papillate hairs inside, lobed with each lobe 1.4 cm in length, reflexed; lateral staminodes 3.0 mm in length × 2.0 mm in width, cup-shaped, glabrous, red; labellum 2.2 cm in length × 3.0 mm in width at apex, linear to spatulate, not lobed, red basally with yellow apex, glabrous; fertile stamen with filament 2.1 cm long, red at throat of corolla becoming yellow distally, glabrous; anther 3.0 mm in length × 2.0 mm in width, without a crest; pollen pale yellow; epigynous glands two, linear; stigma minute, <1 mm across, white, extending slightly beyond anther; ovary trilocular, 2.0 mm in length × 2.0 mm in width, pubescent, white to cream colored. *Fruits* and seeds unknown.

##### Distribution.

Known only from the type locality in Kachin State, upper Myanmar.

##### Ecology.

This species is found in the understory of evergreen forests dominated by members of the family Dipterocarpaceae in the foothills of the Himalayas.

##### Etymology.

Named for Francis Kingdon-Ward, the British plant collector who extensively explored Upper Burma in the first half of the twentieth century and who inspired the authors of this species in their work in Myanmar (Kress et al. 2003, Kress 2009).

##### Other specimens examined.

Known from the type collection in Padaung National Park west of Myitkyina, Kachin State, Myanmar, as well as cultivated material (Smithsonian Botany Research Greenhouse GH-2002-050) collected at the type locality.

#### Larsenianthus careyanus

(Benth.) W. J. Kress & Mood comb. nov.

urn:lsid:ipni.org:names:77107684-1

Plate 1B 

##### Latin

*Basionym: Hitchenia careyana* Benth. (Gen. Pl. 3: 643, 1883).

##### Type.

**India:** “Mts Sillet, Francis de Silva and William Gomez”, 1832. Wallich Cat. Herb. Ind., 6595 (as Curcuma careyana in Wallich [1832]; lectotype [designated here]: K!; isolectotypes: E!, BM!).

##### Description.

*Evergreen herb*, medium-sized to 215 cm tall; rhizome to 2.5 cm in diameter, fibrous, fragrant, inner color with an outer white layer and inner dull white layer, roots 5 mm in diameter, yellow; tubers absent. *Leafy shoots* 10–30, densely clumped, stems 3–4 cm in diameter at base. *Leaves* 7–9 per stem, increasing in size upward, to 40 cm in total length; basal leaf sheaths 3, glossy, dark green with sparse hairs, turning brown; petiole 0–2 mm in length; ligule 7–8 cm in length cm × 4 cm in width, semi-transparent, green turning brown, densely pubescent with short hairs, apex rounded to truncate; lamina 36–60 cm in length × 18–19 cm in width, ovate to elliptic, surface broadly corrugate with prominent veins, dark green, glossy, glabrous, abaxial side dull green, glabrous, midrib with few hairs, margin hyaline, ciliate, base acute, apex acuminate. *Inflorescence* terminal on leafy shoot, erect to 35 cm in height, apical part of peduncle 11–12 cm in length × 1.2–1.8 cm in width clasped by the top two leaf sheaths, light yellow-green, glabrous; spike cylindric, 22–25 cm in length × 6–7 cm in diameter; inflorescence bracts up to 60 per inflorescence, 3–4 lower bracts sterile, individual bracts ovate, 3.5 cm in length × 3 cm in width, green with a broad white edge, glabrous, striate, inside smooth with copious, sticky mucilage, apex mucronate reflexed 90°, margin hyaline; 1–3 adventitious plantlets produced in sterile bracts at base of old inflorescence; cincinnus 1 per bract; flowers mature from base to apex of inflorescence. Bracteoles lanceolate, cymbiform, not tubular, keeled with uneven sides, 25–28 mm in length × 5–15 mm in width, striate, yellow-tan, apex truncate to slightly acute. *Flowers* conspicuous, 4–6 per bract; calyx tubular, 10–18 mm in length, trilobed, white, transparent, glabrous; floral tube 45–52 in length × 1–2 mm in diameter, white, glabrous, deflexing 45° toward rachis in last 10 mm, lobes 3, linear-lanceolate, 15 mm in length × 10 mm in width, greenish-white with pink apex, dorsal lobe recurved 180° against corolla tube, ventral lobes deflexed downward, twisted, almost parallel with labellum; lateral staminodes 2 mm in length × 2 mm in width, suborbicular, white, irridescent, reflexed, apex truncate, pink, glabrous; labellum 24 mm in length mm × 6 mm at the widest, elongate, oblanceolate, white to light purple-pink, iridescent with reflective cells giving a sparkle effect, apex truncate, bidentate at center; fertile stamen with filament 2.3 cm long, forming a 180° arc, ending c. 1 cm above labellum, white; anther 5 mm in length × 3 mm in width, oblong, pollen white; epigynous glands two, cuneate, 5 mm in length × 1 mm in diameter, yellowish; stigma, c. 1 mm long, apical opening with hairs, white, extending 2 mm beyond thecae; ovary trilocular. *Fruits* 2.0 cm in length × 1.0 cm in diameter, capsular, dehiscing into three separate coiled sections; seeds 8–10, 5 mm in length × 3 mm in width, shiny green, enclosed in a thin, tripartite, cocoon-like fused aril.

##### Distribution.

Bangladesh and northeastern India (Assam, Meghalaya, Arunachal Pradesh, Manipur).

##### Ecology.

Larsenianthus careyanus is found in tropical semi-evergreen forest.

##### Etymology.

This species was originally named by Wallich for the botanist Dr. William Carey (1761–1834).

##### Specimens examined.

**India:** Arunachal Pradesh: Lohit Dist., 15 September 1969, A.S. Rao 47859 (CAL!); Tirap F. D., 10 November 1959, R. Seshagiri Rao 20004 (CAL!). Assam: Dulong Reserve Forest, 21 November 1957, G. Panigrahi 11283 (CAL!); Kabakhal Reserve Forest, 9 January 1957, R. Seshagiri Rao 9077 (CAL!); Kakoi Reserve Forest, N. Lakhimpur, 13 May 1966, D.M.Verma s.n. (ASSAM!). Manipur: Thirighat, February 1906, A. Meebold 10817 (CAL!); Thirighat, November 1907, A. Meebold 6237 (CAL!). **Bangladesh:** (India, East Bengal), no date, Griffith 5627 (CAL!); near Sylhet, from material collected by M. Collins and cultivated in the US Botany Research Greenhouses (#02146), 29 July 2003, W. J. Kress 03-7403 (US!).

#### Larsenianthus assamensis

S. Dey, Mood, & S. Choudhury sp. nov.

urn:lsid:ipni.org:names:77107685-1

Fig. 3 Plate 1C 

##### Latin

*Species nova* L. careyano *affinis, a quo planta minore semidecumbenti, inflorescentia minore (circa 8 × 4 cm) ovoidea bracteis coccineis ad margines dentatis, ad bracteam quamque flore fertili solitari et labello angustiore magis roseo differt.*

##### Type.

**India:** Assam: Cachar Dist., Bhaluknala, Barail Wildlife Sanctuary, narrow substream of Lakhicherra, 24°58'50.7" N, 92°46'30.8" E. Cachar tropical semi-evergreen forest, 26 m elevation, August 2007, S. Dey 1012 (holotype: CAL!; isotype: ASSAM!).

##### Description.

*Evergreen herb*, medium-sized to 144 cm tall, rhizome fibrous, elongate in distinct sections, bulbous, white-colored internally; tubers absent. *Leafy shoots* 7–17 per plant, erect or slightly decumbent, base bulbous. *Leaves* 7–12 per shoot, to 35 cm in total length; basal leaf sheaths 3–5, light pink, drying brown, deciduous; petiole 0–7 mm in length; ligule 7 mm in length × 5 mm in width, bilobed, apices rounded; lamina 24–34 cm in length × 5.4–8.5 cm in width, elliptic, dark green and glabrous above, base aequilateral, tip acuminate, midrib and veins depressed, abaxial surface dull green and glaucous. *Inflorescence* terminal on leafy shoot, erect to 14 cm in height; apical part of peduncle 3–5 cm in length × 3–5 mm in diameter, light green-brown, glabrous; involucral bract 4.2 cm in length × 1 cm in width, green, red striated, apex cirrose; 1 adventitious plantlet produced in sterile bracts at base of old inflorescence; spike ovoid to ellipsoid, 5.5–8.5 cm in length × 3.8–4 cm in diameter; inflorescence bracts c. 35 per inflorescence, 2.1–2.8 cm in length × 1–1.3 cm in width, ovate-oblong, concave, loosely clasping, red-veined, margin corrugate and denticulate, apex mucronate, revolute with long hairs, apical bracts smaller, sterile; one cincinnus per bract; bracteole lanceolate, cymbiform, 27 mm in length × 19 mm in width, semi-transparent, white, apex tapered, red. *Flowers* conspicuous, 1–4 per bract (usually only a single flower fertile); calyx tubular, 8–14 mm in length × 5 mm in diameter, trilobed, white, apex pink; floral tube 19–27 mm in length × 2–2.5 mm in diameter, pink-red, glabrous, corolla lobes linear-lanceolate, dorsal lobe 13 mm in length × 2 mm in width and reflexed 90°, ventral lobes 11 mm in length × 2 mm in width and deflexed and parallel to floral tube, orange-red with darker apex; lateral staminodes ovate, 6 mm in length × 3 mm in width, light orange with translucent dots, apex irregular, reflexed; labellum 22–25 mm in length × 2–3 mm in width, elongate, oblong, ‘v’ in cross-section, basal margins thickened, light orange, apex slightly trilobed, outer lobes 1 mm in length, rounded, curved upward toward apex, purplish-white; fertile stamen with filament c. 18 mm in length, arched, greenish purple at base, light orange-white distally, oblong, anther 3 mm in length × 0.5 mm in width, thecae glossy purple; pollen white; epigynous glands two, unequal, largest 3 mm in length × 0.5 mm in width and linear, smallest 2 mm in length × 0.5 mm in width and cylindric, dark purple; stigma 0.5 mm in length, extending 5 mm beyond anther, purplish; ovary trilocular, 2 mm in length × 1 mm in width, obovoid-oblong, unequally 3-lobed, pubescent, purplish. *Fruits* 8–9 mm in length × 4–4.5 mm in width, oblong to ellipsoid, white, capsular, dehiscing into three separate coiled sections, seeds 1–4, c. 3 mm in length × 2 mm in width, obovoid, seeds light to dark violet, enclosed in a cocoon-like arillate membrane.

##### Distribution.

Known only from two locations in the Barail Wildlife Sanctuary. Larsenianthus careyanus is also frequently found in this same region.

##### Ecology.

This species occurs in the understory of tropical semi-evergreen forest in very moist conditions along streams (rainfall 350–400 cm/yr) at 25–150 m in elevation. Associated plants are species of Musa, Curculigo, Begonia, Albizzia, Ampelocissus, Dysoxylum, Laportea, Uncaria, and ferns.

##### Etymology.

Named for the Indian state of Assam where this species is endemic.

##### Specimens examined.

**India:** Assam: Cachar Dist., Lakhicherra, Barail Wildlife Sanctuary, 24°59.053' N, 92°46.525' E. ca. 150 m. elevation, 30 August 2007, S. Dey s.n. (living material only).

#### Larsenianthus arunachalensis

M. Sabu, Sanoj & T.Rajesh Kumar sp. nov.

urn:lsid:ipni.org:names:77107686-1

Fig. 4 Plate 1D 

##### Latin

*Species nova* L. careyano *affinis, a quo inflorescentia centrali in caule bifoliato portata et ad bracteam quamque floribus fertilibus 2–3 differt.*

##### Type.

**India:** Arunachal Pradesh: Lohit Dt.: Lalpani, Hayuliang Road, N 27°56'28.2", E 096°22'21.9", 6 August 2009, E.Sanoj & T.Rajesh Kumar 105640 (holotype: CAL!; isotypes: CALI!, ASSAM!).

##### Description.

*Evergreen herb*, medium-sized to 150 cm tall; rhizome 1.9 cm in diameter, hard, fibrous, slightly aromatic, inner color pale brown; tubers absent. *Leafy shoot* erect; base 2.5–3 cm in diameter. *Leaves* two per flowering shoot, to 120 cm in total length; basal leaf sheaths 4–6, red and green, densely pubescent towards apex; petiole 19.5–31 cm in length, cross section U-shaped, pubescent, green; ligule 9.5–14 cm in length × 2.4–2.7 cm in width, lanceolate, apex attenuate, pubescent abaxially, becoming dry and brown; lamina 56–88 cm in length × 19–25 cm in width, abaxially pale green and densely pubescent with silvery hairs, elliptic, dark green, and glabrous above, veins raised 4–6 mm, margins entire, undulate, hyaline, white tinged, base attenuate, apex long acute, slightly twisted. *Inflorescence* terminal on leafy shoot, erect to 90 cm in height; apical part of peduncle 25–75 cm in length, c. 1.2 cm in diameter, pubescent, pale green; spike elliptic, 14–19 cm in height × 3–3.4 cm in diameter; inflorescence bracts 60–80 per inflorescence, bracts 2.4–2.9 cm in length × 2.6–2.8 cm in width, spirally arranged and tightly imbricate, orbicular to broadly elliptic, cymbiform, free to the base, coriaceous, deep red, base white tinged, margin entire and smooth, glabrous, apex acute to rounded, surfaces pubescent, dense brown hairs toward apex; one cincinnus per bract; bracteoles tubular, longer than bracts, 2.8–3.3 cm in length, unilaterally split 1 cm, apex acute to rounded, deep red, white tinged towards base, densely pubescent with short brown hairs towards apex. *Flowers* conspicuous, 2–4 per bract, 2–8 flowers open simultaneously on inflorescence; calyx tubular, 16–17 mm in length × c. 3 mm in width, apex trilobed, unilaterally split 5–6 mm, pale red, white towards base, pubescent with denser hairs towards apex, membranous, translucent; floral tube 3.2–3.3 cm in length × *c*. 3.5 mm in diameter at opening, red, lobed with each lobe 15–17 mm in length, oblanceolate, dorsal lobe reflex, sparsely pubescent externally with scattered unicellular branched hairs inside, lateral lobes glabrous; lateral staminodes *c*. 4 mm in length × 3.5 mm in width, orbicular to broadly elliptic, white with pale red tinge, revolute; labellum 25–28 mm in length × 2.5–3 mm in width, narrowly oblong in first two-thirds and oblanceolate distal third, semi-spathulate, red to creamy yellow towards base and orange-yellow towards acute, beak-like apex; fertile stamen with filament 2.4–2.6 cm in length, red becoming creamy-yellow near anther, arching like a fish-hook; anther *c*.3 mm in length × 2 mm in width, creamy yellow, glabrous; epigynous glands two, oblong, white, 2.5–3.0 mm in length; stigma *c*. 0.5 mm wide, white, bulbous, margins ciliate, exserted 2–2.5 mm from the middle of the anther; ovary trilocular, *c*. 3 mm in length × 2.5 mm in diamter, tomentose, pale red. *Fruits* and seeds unknown.

##### Distribution.

Larsenianthus arunachalensis is narrowly endemic in Arunachal Pradesh, India, and is known only from the type locality. It is highly endangered due to various anthropogenic activities.

##### Ecology.

This species grows in sandy soil above 1,400 m in elevation amidst thick clumps of wild species of Musa.

##### Etymology.

The specific epithet “arunachalaensis” is derived from the name of the state in northeast India from where the type specimen was collected.

##### Specimens examined.

Known only from the type specimen.

## Supplementary Material

XML Treatment for Larsenianthus

XML Treatment for Larsenianthus wardianus

XML Treatment for Larsenianthus careyanus

XML Treatment for Larsenianthus assamensis

XML Treatment for Larsenianthus arunachalensis
